# IL-6, TNF-α and IL-12p70 levels in patients with colorectal cancer and their predictive value in anti-vascular therapy

**DOI:** 10.3389/fonc.2022.997665

**Published:** 2022-09-26

**Authors:** Jingxian Zheng, Xiaojie Wang, Jiami Yu, Zhouwei Zhan, Zengqing Guo

**Affiliations:** ^1^ Department of Medical Oncology, Clinical Oncology School of Fujian Medical University, Fujian Cancer Hospital, Fuzhou, China; ^2^ Fujian Key Laboratory of Translational Cancer Medicine, Fujian Cancer Hospital, The Affiliated Cancer Hospital of Fujian Medical University, Fuzhou, China; ^3^ Fujian Provincial Key Laboratory of Tumor Biotherapy, Fujian Cancer Hospital, The Affiliated Cancer Hospital of Fujian Medical University, Fuzhou, China

**Keywords:** IL-6, colorectal cancer, tumor necrosis factor-alpha, il-12, anti-vascular therapy

## Abstract

We aimed to analyze the levels of interleukin-6 (IL-6), tumor necrosis factor-α (TNF-α) and interleukin-12 (IL-12p70) in colorectal cancer and evaluate the predictive significance of clinical efficacy of patients with colorectal cancer treated with anti-vascular therapy combined with chemotherapy. A retrospective study of 162 patients with colorectal cancer in Fujian Medical University Hospital was conducted from January 2019 to December 2020. A comparative analysis of the levels of IL-6, TNF-α and IL-12p70 between the two groups were studied. The relationship between the levels and the clinical characteristics of patients was observed; the factors affecting the levels of IL-6, TNF-α, and IL-12p70 in colorectal cancer patients were analyzed, and the predictive validity of the efficacy of anti-vascular therapy was evaluated. We observed that the individual expression levels of IL-6, TNF-α and IL-12p70 in the patients with colorectal cancer are related to lymph node metastasis, TNM staging, and degree of differentiation (P<0.05); however, they are irrelevant to the age, sex, and tumor location of patients with colorectal cancer (P>0.05). The multiple stepwise regression analysis indicates that lymph node metastasis and TNM staging are independent risk factors that correlate with IL-6 and IL-12p70 levels in colorectal cancer patients (P<0.01). The degree of differentiation was found to be an independent risk factor connected to TNF- α levels of patients with colorectal cancer. The change of IL-12p70 level could predict the validity of anti-vascular treatment for advanced colorectal cancer. When evaluated for combined expression, IL-6 and IL-12p70 in patients with colorectal cancer closely related to lymph node metastasis and TNM staging. IL-12p70 can be used as a predictor of anti-vascular therapy with colorectal cancer.

## 1 Introduction

The incidence of colorectal cancer is increasing in China (1). Early symptoms are not obvious and there is a lack of convenient and effective specific screening indicators (2). Most patients are already in the advanced stage when they undergo treatment (3). Cytokines such as tumor necrosis factor (TNF) and interleukins (ILs) are closely associated with the differentiation and proliferation of tumor cells and the formation of tumor neovascularization (4), which has an important impact on the progression of patients with tumor. They can be used as a reference index for the clinical outcome and follow-up of patients with colorectal cancer (5). The progression and metastasis of tumors depend on tumor blood vessels. VEGF, which is known to be the only growth factor acting specifically on vascular endothelial cells and directly involved in inducing tumor angiogenesis, strongly induces vascular growth. The over-expression of VEGF itself and its receptor is closely associated with tumor growth, invasion, and metastasis (6).

Tumor angiogenesis is controlled by pro-angiogenic and anti-angiogenic factors, which are secreted by tumor cells, stromal cells, inflammatory cells, or other circulating cells. The balance of cytokines and chemokines affects the efficacy of VEGF inhibitors (7, 8). In addition, VEGF inhibitors, which curb the secretion of vascular endothelial growth factor and complex interactions in the tumor micro-environment, affect the secretion levels of the above-mentioned various factors (9, 10). The baseline expression levels of cytokines and angiogenic factors affect drug activity that can be eventually utilized to predict the beneficiaries, determine the best drug dose, and clarify the mechanism of drug resistance. According to published studies, circulating endothelial cells, soluble VEGF receptor 2, and VEGF are all blood-based biomarkers and have been certificated to be utilized for evaluation of the efficacy of multiple VEGF receptor inhibitors (11, 12). It has been suggested that the baseline levels of circulating VEGF can be used to predict the clinical benefit or tumor response of these drugs, but whether cytokines can be used as a predictor of treatment efficacy needs further verification (13). Multiple technologies provide a non-invasive and convenient method to detect the expression of multiple cytokines based on a small amount of plasma, which offers the possibility of exploring the predictive potential of cytokines (14).

The first-line conventional therapy for metastatic colorectal cancer is bevacizumab (15). It is a re-combined humanized monoclonal antibody directed against VEGF-A. However, the effective rate is only about 60% (16). We selected 162 patients with colorectal cancer from Fujian Medical University Hospital to analyze the correlation between cytokine expression and clinical characteristics in patients at various stages, further analyzing the changes of cytokines before and after treatment of Bevacizumab. The study also analyzed the correlation of tumor regression of patients with late colorectal cancer and initially exploring the predictive significance of cytokines for the efficacy of treatment.

## 2 Materials and methods

### 2.1 Case selection

The study included 196 cases in Fujian Tumor Hospital between January 2019 and June 2020. The study was approved by the Ethics Committee of the Fujian Medical University (10-2018) and informed consent was obtained from all patients.

#### Inclusion criteria

All patients were pathologically diagnosed as suffering from colorectal cancer. Stage I-III patients were treated with radical surgery and stage IV patients should meet the following conditions: (1) clinically, there are measurable lesions; (2) KPS equal to 80 or more; aged 18 years old or above; expected survival time above 3 months; (3) good bone marrow reservation function, normal heart, liver and kidney function and blood coagulation function; (4) no history of traumatic surgery within 4 weeks and no brain metastasis, with imaging showing no important vascular invasion, no uncontrollable hypertension (systolic blood pressure is 150 mmHg and/or diastolic blood pressure is below 100 mmHg), no active cardiovascular and cerebrovascular disease within 6 months; (5) at least after the treatment (3 -week plan for 2 cycles, 2 -week plan for 3 cycles).

### 2.2 General information

162 cases met the selection criteria, including 77 males and 85 females aged 53-78, the medium age of which was 65.16 ± 4.25; 84 cases with left and right colon and sigmoid colon cancer, 78 cases with rectal cancer, 77 cases with tubular adenocarcinoma and 84 cases with mucinous adenocarcinoma; 52 cases with medium/high differentiation, 110 cases with poor differentiation; 113 cases under the treatment of radical surgery, 12 cases palliative surgery; 25 cases on the first-line chemotherapy on the basis of oxaliplatin; 24 cases on the foundation of irinotecan; 74 cases in stage I-II, 88 cases in stage III-IV, and 49 cases in stage IV.

### 2.3 Method

#### 2.3.1 Treatment plan

The dose of Bevacizumab was 5mg/kg, intravenous drip for 30 to 90 minutes, once every 2 weeks. The dose of Bevacizumab was 7.5mg/kg, intravenous drip for 30 to 90 minutes, once every 3 weeks. FOLFOX6 regimen: Oxaliplatin (OXA) 100mg/m2, day 1: 2 hours of intravenous drip, Levoleucovorin (LV) 400 mg/m2, intravenous infusion for 2 hours; 5-fluorouracil (5 -FU) 400mg/m2, intravenous drip, the first day; 5-FU 2400~3000mg/m2, continuous pumping for 46 hours. Every 14 days constituted a cycle. FOLFIRI regiment: Irinotecan (CPT-11) 180 mg/m2, intravenous infusion for 90 minutes for the first day; LV 200mg/m2, intravenous infusion for 2 hours for the first day; LV 400 mg/m2, intravenous infusion, the first day; 5-FU 2400mg/m2, pumped continuously for 46 hours. 14 days constituted a period. XELOX regiment: Oxaliplatin (OXA) 100 mg/m2, intravenous drip for 2 hours on the first day; capecitabine (CAP) 1000 mg/m2 for the first fourteenth days (17). There were 21 days for a period. The chemotherapy regiment was administered simultaneously with bevacizumab.

#### 2.3.2 Extracting peripheral blood from the right arm of all patients before treatment

Treatment of 3 weeks and 6 weeks, (1) taking heparin or EDTA anticoagulant whole blood (100 μl/part), set 12 × 75 mm in the special plastic test tube. (2) 20 μl of specific fluorescent monocompans was added per tube; the other with the transfection of a fluorescently labeled anti-parallel and staining at room temperature for 15 minutes. (3) Dissolve red blood cells with the help of a Q-Prep instrument, subsequently stabilizing and fixing white blood cells and locating for 5 minutes. (4) Detect with the upper flow cytometry.

#### 2.3.3. Indirect fluorescent staining of micro whole blood

(1) taking heparin or EDTA anticoagulated whole blood (100μl/part) and locating in a 12×75mm special plastic test tube. (2) Adding 50μl of specific monoclonal antibody (primary antibody) and incubating for 30minutes at room temperature. (3) Depositing on the Q-PREP instrument to dissolve red blood cells, stabilizing and fixing white blood cells. (4) Centrifuging (800-1000 rpm, 5 min) to discard the serum, washing the cells twice with PBS. (5) Adding 50μl of fluorescent (FITC or PE)-labeled secondary antibody, locating for 30minues in the darkness at room temperature. (6) Finally, detecting with the upper flow cytometry.

### 2.4. Efficacy evaluation criteria

The short-term efficacy evaluation criteria of solid tumors refers to the RECIST 1.1 version (18), classified as complete remission (CR), partial remission (PR), disease progression (PD), stable disease (SD). Calculate the response rate (RR) with CR+PR and disease control rate (DCR) with CR+PR+SD. Adverse reactions were evaluated in accordance with the General Toxicity Criteria (NCI-CTC) version 3.0 set by the National Cancer Institute. (Because the median survival period has not reached as of the follow-up endpoint of this study, progression-free survival (PFS) is used as the main observational indicator).

### 2.5. The follow-up

In this study, telephone and imaging follow-up were used, with no loss of patients. Progression-free survival (PFS) is the time from the beginning of treatment to disease progression or death, while overall survival (OS) is that from the beginning of treatment to death or the last follow-up. The deadline for follow-up is November 31, 2020, with the follow-up time 23 months and the median follow-up time 11 ± 0.2 months.

### 2.6. Statistical method

We used SPSS17.0 statistical software to analyze data, Kaplan-Meier method to perform the survival analysis and the Pearson card testing to correlate analysis of data. The difference value (p <0.05) is statistically significant.

## 3 Results

### 3.1 Single factor analysis result of cytokine expression levels in patients with colorectal cancer

Our initial goal was to assess levels of cytokines individually in colorectal cancer patients. We started with the evaluation of levels of IL-6, TNF-α and IL-12P7, and observed that IL-6 levels are associated with lymph node metastasis and the TNM staging in patients with colorectal cancer (t = 6.705, -4.224, P <0.05). TNF-α levels, on the other hand, related to the degree of differentiation (t = -213, P <0.05) while IL-12P7 levels correlated with lymph node metastasis and TNM staging of patients (t = -2.713, 2.681, P <0.01). Also, IL-6, TNF-α and IL-12P7 levels did not correlate with age, gender, tumor site and histological classification (P> 0.05) (**Table 1**).

**Table 1:** The single factor analysis result of cytokine expression levels in patients with colorectal cancer.

Item		Number	IL-6 (mg/L, X ± S)	TNF-α (mg/L, X ± S)	IL-12 (pg/ml, X ± S)
Gender	Male	77	6.48 ± 1.19	2.38 ± 0.87	1.69 ± 0.38
	Female	85	6.14 ± 2.15	2.17 ± 0.65	1.67 ± 0.39
	t		1.476	1.203	1.103
	p		0.158	0.243	0.689
Age	≥65years	77	6.81 ± 1.25	2.26 ± 0.71	1.68 ± 0.35
	<65 years	85	6.47 ± 2.06	2.14 ± 0.82	1.67 ± 0.42
	t		1.503	1.07	1.139
	p		0.131	0.310	0.130
Location	Colon	84	5.78 ± 2.09	2.16 ± 0.82	1.66 ± 0.41
	Rectum	78	6.17 ± 1.28	2.24 ± 0.70	1.71 ± 0.36
	t		-1.342	-0.677	-0.784
	p		0.143	0.497	0.434
Lymph node metastasis	Yes	104	6.87 ± 1.19	2.35 ± 0.77	1.62 ± 0.35
	No	58	5.10 ± 2.17	2.23 ± 0.68	1.79 ± 0.45
	t		6.705	1.527	-2.713
	p		<0.001	0.129	0.007
Degree of differentiation	High/Medium	52	6.41 ± 2.36	2.00 ± 0.58	1.63 ± 0.38
	Low	110	6.62 ± 1.32	2.29 ± 0.82	1.71 ± 0.39
	t		-1.163	-2.213	-1.273
	p		0.217	0.028	0.206
Histological classification	Tubular cancer	77	6.53 ± 1.66	2.29 ± 0.74	1.74 ± 0.40
	Mucinous cancer	85	5.97 ± 1.91	2.11 ± 0.78	1.63 ± 0.37
	t		1.979	1.537	1.742
	p		0.051	0.126	0.084
TNM staging	I-II	74	5.59 ± 2.01	1.97 ± 0.71	1.77 ± 0.39
	III-IV	88	6.77 ± 1.43	2.19 ± 0.76	1.61 ± 0.38
	t		-4.224	-1.610	2.681
	p		<0.001	0.106	0.008

### 3.2 Multi-factor analysis

After performing the evaluation of IL-6, TNF-α and IL-12P7 individually, we next checked for their combined expression. Multi-factor analysis of IL-6, TNF-α and IL-12P7 levels in patients refers to three factors (lymph node metastasis, historical score and TNM staging) as the argument, which uses the expression of IL-6, TNF-α and IL-12P7 as the dependent variable by multi-step regression analysis. The results indicated that lymph node metastasis and TNM staging are both independent risk factors connected to IL-6 and IL-12P7; the degree of differentiation being an independent risk factor (P <0.01) connected with TNF-α level (P <0.01) (**Tables 2**–**4**).

**Table 2:** Multi-factor analysis of the IL-6 level in patients with colorectal cancer.

Factor	Non-Standardized Coefficient	Standard Error	Standardized Coefficient	T-Value	P-Value
(Constant)	5.558	.722		7.699	<0.001
Lymph node metastasis	-1.484	.254	-.394	4.960	<0.001
Degree of differentiation	.145	.260	0.101	1.261	0.215
TNM staging	.933	.239	0.257	3.906	<0.001

**Table 3:** Multi-factor analysis of the influencing factors of the TNF-α level in patients with colorectal cancer.

Factor	Non-Standardized Coefficient	Standard Error	Standardized Coefficient	T-Value	P-Value
(Constant)	1.851	0.345		5.369	<0.001
Lymph node metastasis	-0.148	0.121	-0.109	-1.105	0.213
Degree of differentiation	0.365	0.114	0.238	3.196	0.002
TNM staging	0.155	0.124	0.095	1.247	0.214

**Table 4:** Multi-factor analysis of the IL-12P7 level in patients with colorectal cancer.

Factor	Non-Standardized Coefficient	Standard Error	Standardized Coefficient	T-Value	P-Value
(Constant)	1.404	.181		7.750	<0.001
Lymph node metastasis	.135	.071	.145	1.885	0.041
Degree of differentiation	.061	.067	.070	0.9044	0.367
TNM staging	.158	.067	.183	2.372	0.019

### 3.3 Patient prognosis

**Figure 1:**
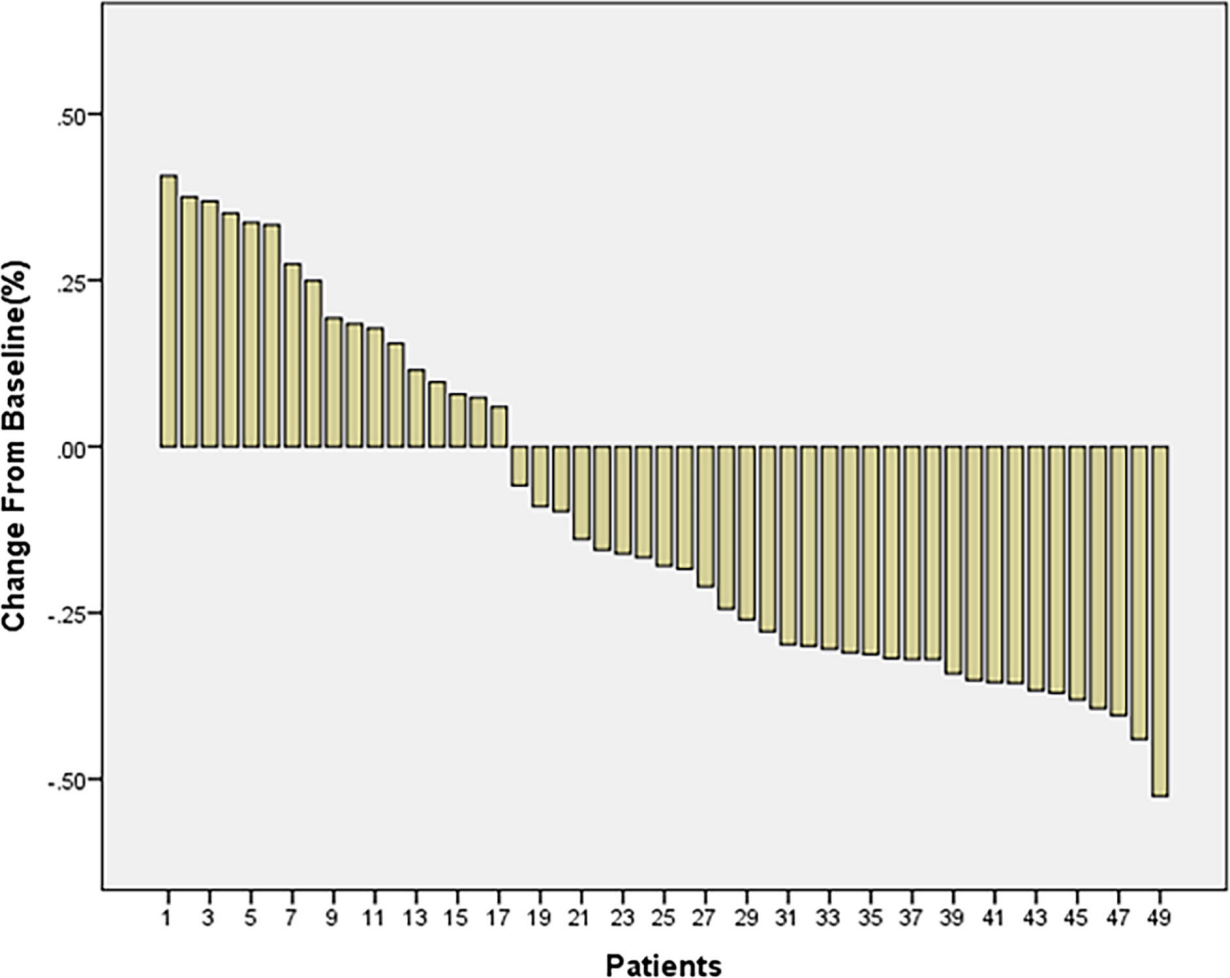
The treatment of tumor patients in stage IV.

Next, we checked the patient prognosis. The average treatment time for patients was 52 days (14 to 84 days) in stage IV. Among the 49 patients, 32 had shrinkage of their tumors: 17 had tumor progression. The treatment efficiency was found to be 65.3%, the aggrandizement range being 6% -41% and the reduction range being 6% -53% (**Figure 1**).

### 3.4 IL-12p7

**Figure 2:**
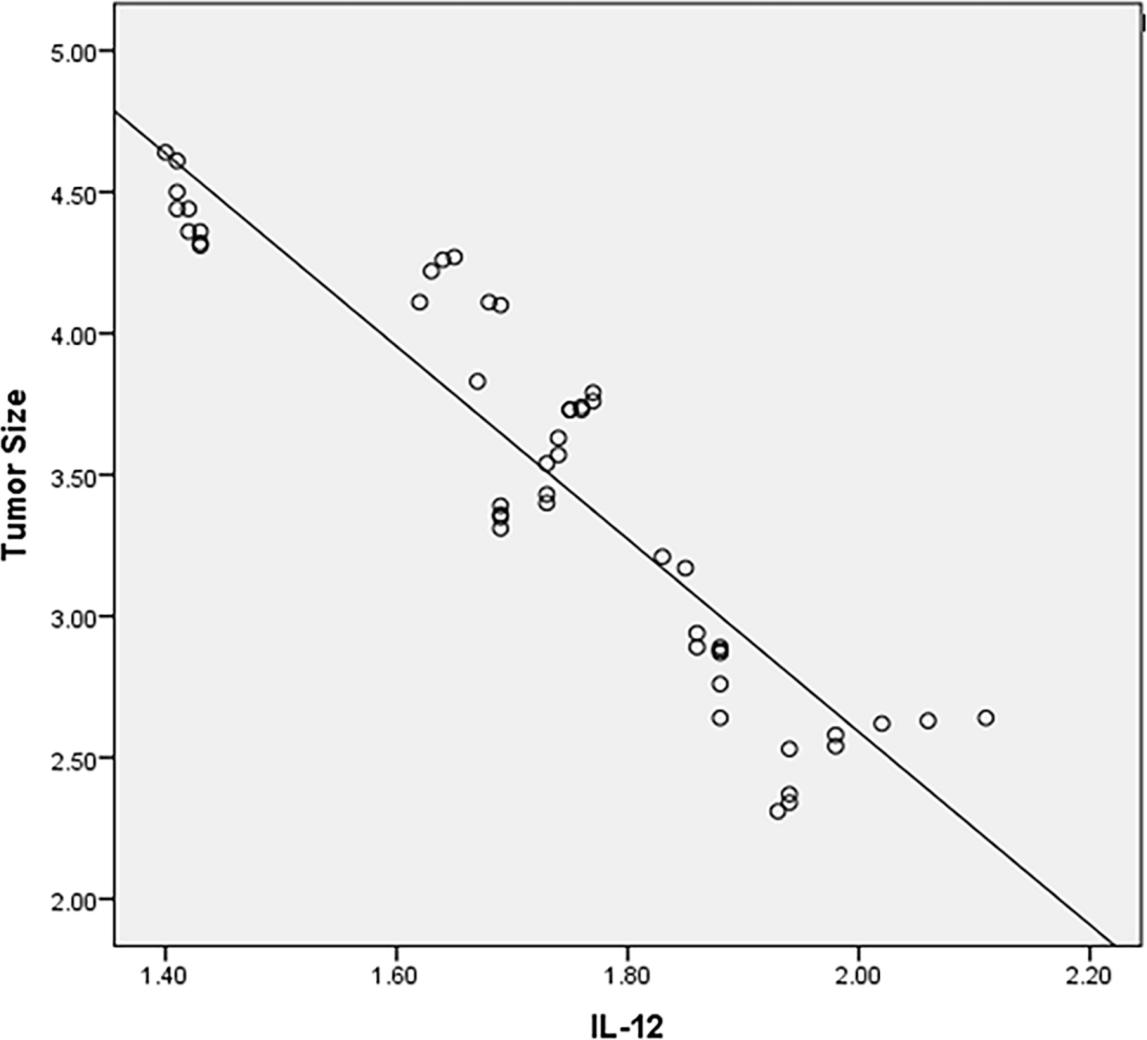
The correlation between the variation and efficacy before and after the treatment.

The baseline level of IL-12p7 in patients was found to be associated with tumor reaction. We observed that, under IL-12P7 treatment, the variation is significantly associated with tumor reduction, and both are negatively correlated, which can be the marker for predicting treatment efficacy (**Figure 2**).

## 4. Discussion

Inflammatory factors are mainly affected by changing cellular microenvironments to promote cell proliferation and induce cellular carcinogenic or tumor suppressor gene mutation (19). Many inflammatory factors exist in the tumor microenvironment, including IL-1, IL-6, IL-12, IL-17, TNF-α and TGF-β (20–22). They not only play the role of raising inflammatory cells to tumor parts; zooming in inflammation but also promoting tumor cell growth and metastasis; meantime accelerating tumor blood vessel and lymphatic production. Tumor angiogenesis and inflammatory factors promote each other, forming malicious tumor microenvironment (23). Therefore, by exploring the interrelation between anti-angiogenesis and cytokines, it is desirable to find possible anti-angiogenic effective biomarkers. This paper examines the interrelation among IL-6, TNF-α and IL-12P7 secreted by Th1 and Th2 with the attempt to indicate the relationship between tumor angiogenesis and tumor immunocellular environmental reprogramming.

IL-6 can induce the formation of signals, transduction protein and transcriptional activation factor-3 and makes joint efforts in TGF-β1 generation and epithelial conversion, inhibiting T-cell proliferation and killing effect and activating Treg, which plays the key role in the immunization of colorectal cancer and is related to the adverse prognosis (24). TNF-α has the function of modifying ribosome C (for biochemistry) which can improve the mismatched risk of chromosome DNA and inhibit the activity of mismatched repaired factor CYC. In addition, TNF-α can also increase the malignancy and adhesion of tumor cells to vascular endothelial or lymphatic endothelium, and tumor cells through lymphatic or blood-transfer risk (25, 26).

Our results show that the IL-6 levels of patients with neutral colorectal cancer in late clinical staging were higher (P <0.05) and the TNF-α levels of patients with low division were higher (P<0.05). In terms of installment, IL-12 levels of patients with no lymph node metastasis were higher (P<0.05). Studies have found that the level of expression of related cytokines of patients whose tumor diameters are longer has increased significantly, but the influence of cytokines on tumor diameters needs further examination (27). It is believed that the possible mechanism of cytokines affected the formation of new blood vessels, which therefore had an influence on the blood flow perfusion of local tumor lesions (28), however, it needs further study. According to the analysis of risk factor, we can also draw the conclusion that lymph node metastasis and TNM staging are statistically relevant to IL-6 and IL-12P7; in addition, the statistical correlation of degree of differentiation with TNF-α further suggests IL-6, TNF-α and IL-12P7 are closely related to the condition of patients. The above indicators may be involved in the progression of malignant tumors. By analyzing the factors for high expressions of IL-6 and TNF-α, it can promote transcriptional activity of tumor cell, promote the change of tumor cell biological characteristics, improve the infiltration of tumor cells to intestinal mucosal muscle layers or subcutaneous tissue, and further affect the occurrence and development of colorectal cancer.

The baseline level of IL-12 has the best correlation with tumor reaction, which is likely to be labeled as an important predictive indicator and is the main regulator of the Th1 immune responses and the known vascular production inhibitor. As far as we know, this is the reaction predictor of the cyclic IL-12 which accepts an anti-angiogenic TKI of patients. In summary, levels of IL-6, TNF-α and IL-12P7 in plasma of patients with advanced colorectal cancer increased. In addition, IL-6 and IL-12P7 are associated with TNM staging and lymph node metastasis, while TNF-α is related to differentiation. The elevation of the expressional level can predict effective outcome of antivascular therapy of colorectal cancer. Preliminary findings of this study are that there is correlation between plasma cytokines and advanced colorectal cancer, which may be a predictive indicator of anti-visional therapeutic efficacy. However, this study is to take a further clinical trial in the future.

## Data availability statement

The original contributions presented in the study are included in the article/supplementary material. Further inquiries can be directed to the corresponding author.

## Ethics statement

The studies involving human participants were reviewed and approved by Ethics Committee of the Fujian Medical University. The patients/participants provided their written informed consent to participate in this study.

## Author contributions

JZ, JY, and ZZ collected patient data, JZ, XW, JY, and ZZ analyzed data and prepared manuscript, ZG supervised study and arranged experiments. All authors contributed to the article and approved the submitted version.

## Funding

Startup Fund for scientific research, Fujian Medical University (No. 2018QH1224); Fujian Provincial Health Technology Project (No. 2019-2-6); Fujian Provincial Health Technology project (No. 2020J011111); Fujian Innovation project (No. 2019-CX-4).

## Conflict of interest

The authors declare that the research was conducted in the absence of any commercial or financial relationships that could be construed as a potential conflict of interest.

## Publisher’s note

All claims expressed in this article are solely those of the authors and do not necessarily represent those of their affiliated organizations, or those of the publisher, the editors and the reviewers. Any product that may be evaluated in this article, or claim that may be made by its manufacturer, is not guaranteed or endorsed by the publisher.
